# State-of-the-art methodologies used in preclinical studies to assess left ventricular diastolic and systolic function in mice, pitfalls and troubleshooting

**DOI:** 10.3389/fcvm.2023.1228789

**Published:** 2023-08-07

**Authors:** Gianluigi Pironti

**Affiliations:** ^1^ Cardiology Research Unit, Department of Medicine, Karolinska Institutet, Stockholm, Sweden; ^2^ Department of Physiology and Pharmacology, Karolinska Institutet, Stockholm, Sweden

**Keywords:** cardiovascular diseases, heart failure, systolic function, diastolic function, echocardiography, MRI, PV loop analysis

## Abstract

Cardiovascular diseases (CVD) are still the leading cause of death worldwide. The improved survival of patients with comorbidities such as type 2 diabetes, hypertension, obesity together with the extension of life expectancy contributes to raise the prevalence of CVD in the increasingly aged society. Therefore, a translational research platform that enables precise evaluation of cardiovascular function in healthy and disease condition and assess the efficacy of novel pharmacological treatments, could implement basic science and contribute to reduce CVD burden. Heart failure is a deadly syndrome characterized by the inability of the heart to meet the oxygen demands of the body (unless there is a compensatory increased of filling pressure) and can manifest either with reduced ejection fraction (HFrEF) or preserved ejection fraction (HFpEF). The development and progression of HFrEF is mostly attributable to impaired contractile performance (systole), while in HFpEF the main problem resides in decreased ability of left ventricle to relax and allow the blood filling (diastole). Murine preclinical models have been broadly used in research to understand pathophysiologic mechanisms of heart failure and test the efficacy of novel therapies. Several methods have been employed to characterise cardiac systolic and diastolic function including Pressure Volume (PV) loop hemodynamic analysis, echocardiography and Magnetic Resonance Imaging (MRI). The choice of one methodology or another depends on many aspects including budget available, skills of the operator and design of the study. The aim of this review is to discuss the importance of several methodologies that are commonly used to characterise the cardiovascular phenotype of preclinical models of heart failure highlighting advantages and limitation of each procedure. Although it requires highly skilled operators for execution, PV loop analysis represents the “gold standard” methodology that enables the assessment of left ventricular performance also independently of vascular loading conditions and heart rate, which conferee a really high physiologic importance to this procedure.

## Introduction

The ability of the left ventricle (LV) to contract and propel blood to the rest of the organs is defined as systolic function. The percent of blood ejected from the left ventricle during cardiac cycle (ejection fraction, EF) is commonly measured to assess contractility performance of the heart. Diastolic function encompasses for relaxation and filling of cardiac chambers to enable an adequate blood volume and maintain normal cardiac output. Myocardial relaxation significantly influences early ventricular filling, as well as passive end-diastolic ventricular stiffness, which impacts on late ventricular filling.

Heart failure with impaired systolic function (HFrEF) is frequently accompanied by left ventricular diastolic dysfunction, whereas heart failure with impaired diastolic function (HFpEF) also occurs in the absence of (or eventually precedes) severe systolic dysfunction. In the presence of either preserved or minimally depressed ejection fraction, diastolic dysfunction is the main determinant in patients with HFpEF. Conditions found to be associated with isolated HFpEF include hypertensive cardiac hypertrophy, atrial fibrillation, diabetes mellitus, obesity, metabolic syndrome and aging (1, 2).

Abnormal relaxation and/or increased myocardial stiffness of LV could cause diastolic dysfunction and eventually lead to elevated left ventricle filling pressures and manifestation of HF symptoms. Assessment of diastolic dysfunction is needed to characterize the phenotype of HFpEF, which currently accounts for nearly half of the HF patients, and its prevalence continues to rise due to the increasingly aged society and survival of patients with comorbidities such as type 2 diabetes, hypertension, and obesity (3). Although the prevalence of HFpEF keeps rising, the therapeutic and prevention options are limited or not effective, due to a lack of clear mechanism or pathophysiological understanding, patient heterogeneity, and underdiagnosis (4–7).

Preclinical animal research is the cornerstone of the development of preventive and treatment strategies for cardiovascular disease. A broad variety of preclinical HF models have been extensively employed in basic cardiovascular research to investigate novel pathophysiologic mechanism or test the effect of novel therapies (8, 9). Myocardial infarction (MI) or transverse aortic constriction (TAC), induced by permanent ligation of coronary artery or banding of ascending aorta to induce pressure overload respectively, represent established models of HFrEF (10–15). In absence of a direct myocardial injury, the impaired contractile performance of the heart has also been described in models of rheumatoid arthritis where chronic inflammation was associated with systolic dysfunction (16) while the acute phase of rheumatoid arthritis is characterized by impaired myofibrils cross-bridges formation in response to elevated intracellular Ca^2+^ (17). Several transgenic models have been used to identify important causes of heart failure or its progression and to identify putative targets for therapy (8, 9). Epigenetic factors play also a key role in cardiac remodelling and left ventricle dysfunction is observed in female offspring of obese mothers exposed to high androgen levels (18). Several cardiometabolic disease have been used as HFpEF models presenting impaired ventricle blood filling function rather than decreased contractility (19, 20). It is important to consider that all conditions that are frequently associated with HFpEF (e.g., hypertension, diabetes mellitus, obesity, kidney failure and aging) can be easily recreated in animal models (21) and cardiometabolic dysfunction might remain hidden and can only be revealed through a proper cardiovascular phenotype characterization. Therefore, in preclinical studies it is important to assess both systolic and diastolic cardiac function in all these models in order to have a comprehensive view of novel pathophysiological mechanism responsible of heart failure. Moreover, understanding the utility of different methodological approach and correct interpretation of cardiovascular outcomes will be necessary to establish a standardized translational research platform that allow precise evaluation of cardiovascular function in different pathophysiological stage of disease and evaluate efficacy of pharmacological treatment.

The scope of this review is to provide a detailed overview of state-of-the-art methodologies used in preclinical research for cardiovascular phenotype characterization aimed to implement the insights in this field and reduce cardiovascular disease burden.

Echocardiography, magnetic resonance imaging and Pressure Volume (PV) loop analysis are all valid tools to proper characterize the cardiovascular phenotype of preclinical models. However, the choice of one method or another depends on resources, skills of the operator, time available and study design (Table 1, comparison of different procedures). In this article we will discuss the advantage and disadvantages of different procedures, with focus on PV loop analysis, which represents the “gold-standard” method for the assessment of ventricular systolic and diastolic performance independently of ventricle load condition.

**Table 1:** Comparison between different methodologies used to assess cardiac performance.

	PV loop	Echocardiography	MRI
Cost (euros)	30–40 thousand	300–400 thousand	1–3 million
Size	Portable, small size (it requires a laptop for the software)	Portable and easy to move as it stands on wheels	Not portable, requires a dedicated room due to big size of the equipment
Time of execution per mouse (minutes)	30–120	20–40	90–120
Advantages/Pros	Provides information of pressure and volume changes in real time. Allows assessment of load-independent systolic and diastolic function, i.e., end systolic and end diastolic pressure volume relationship. Allows measurements of several hemodynamic parameters pre and post drug administration within the same mouse.	Allows simultaneous measurements of physiologic parameters. Allows measurements of heart dimensions: chambers, pericardium, valves, strain. Non-invasive procedure. It can be used for serial measurements.	Allows measurements of cardiac fibrosis and tissue perfusion *in vivo*. Allows measurements of physiologic parameters and heart dimensions (chambers, pericardium, valves, strain). High spatial resolution and high accuracy and reproducibility. Non-invasive procedure. It can be used for serial measurements
Disadvantages/Cons	Invasive, terminal procedure, it does not allow serial measurements. Requires good surgical skills of the operator. Cannot give direct information about heart dimension. Possible artifacts due to outflow track obstruction or catheter entrapment in papillary muscles. Heart rate should be between 400 and 600 bpm and mean blood pressure should be more than 90 mmHg to ensure physiological relevance.	Technical variability due to type of anaesthesia used and skills of the operator. Acclimation is needed for serial measurements of the same mouse. Variability due to operator skills and type of anaesthesia used Pulse wave doppler artifacts due to high HR or lower transducer frequency (i.e., fusion of E/A waves). Load dependent data. Heart rate should be between 400 and 600 bpm to ensure physiological relevance.	Time consuming procedure. Not commonly used to assess diastolic function. Cardiac gating necessary. Signal to noise ratio limitation, risk of motion artifacts. Uses contrast agents. Load dependent data. Heart rate should be between 400 and 600 bpm to ensure physiological relevance.

## Echocardiography screening

Cardiovascular function is routinely assessed by echocardiographic ultrasound imaging in patients, while murine myocardial characterization requires high spatial and temporal resolution in order to maintain a physiological state. Small animal size (mouse, ∼20 g), orientation of the heart, and high heart rates (HRs, 400–600 beats/min) are some of the challenges that have been overcome lately via high-frequency transducers (up to 70 MHz), improved signal processing, and superior imaging frame rates (700 frames/s), providing superior resolution (∼30 μm) and image uniformity throughout the field with novel post-acquisition analysis, although considerable training in hands-on imaging is required.

Echocardiography screening allows non-invasive assessment of systolic function and cardiac morphology, however diastolic function evaluation through echocardiography becomes challenging due to the high heart rate (400–600 bpm) and the difficulty to orient the echo probe in mice using four chambers view of the heart. Detailed protocol for accurate echocardiography screening in murine models has been described in recently published guidelines by Zacchigna et al. (22).

The ultrasound imaging of the heart is a non-invasive method to screen for cardiac disfunction, that can be executed in relatively less time compared to other methodologies. However, it requires an advanced and costly echocardiography machine that include Pulse Wave and Tissue Doppler modules in order to trace the movement of targets (blood cells in Pulse Wave Doppler or myocardium in Tissue Doppler) for characterization of diastolic functions. This latter requires an accurate four chambers projection during the echo imaging, which is definitely not easy to obtain due to the small size of the animals. Most of the echocardiography imaging is performed using a 2-D view of the heart (B-Mode) or single axis scanning (M-mode) displayed over time for assessment of cardiac dimension, visualization of anatomic structures or evaluation of systolic functional parameters (e.g., LV fractional shortening). The limitation of LV volumes and EF calculation using M-mode is linked to the assumption of a spherical or ellipsoid shape of the heart (referred as D3 formula or Teichholz formula, described below), which is not accurate and leads to greater errors in pathological models with aggravated heart remodelling (i.e., MI or TAC).

Alternatively, speckle-tracking echocardiography (strain imaging) is used to track the motions of the myocardium by analysing B-mode images of the “speckles” that deforms over time through a postprocessing computer algorithm (23). This technique is also referred as deformation imaging since it tracks the motion of the heart by calculating the amount of its deformation over time, which can be expressed as velocity (first derivation of the strain rate) and implement the functional information. In clinical studies strain imaging is commonly used due to the higher sensitivity for changes in myocardial contractility and regional wall motion (24). Technological implementation of modern ultrasound machines enabled the use of speckle tracking echocardiography also in preclinical studies (25, 26). An aspect that requires more investigation in preclinical studies is the assessment of the twisting and untwisting rates during systolic contraction and diastolic relaxation respectively. In fact, the base and the apex of the heart rotate in opposite directions due to the arrangement of myofiber sheets within the epicardium and endocardium wall resulting in the upward swing of the apex during systole which leads to accumulation of strain energy that is released during isovolumic relaxation. Hypertrophy, fibrosis and wall stiffening that mostly characterize hypertrophic cardiomyopathy causes myofibers disarray and impaired twisting properties and elastic recoil (27), therefore implementation of current methodologies with torsion analysis could simplify the detection of early diastolic dysfunction in murine preclinical models (28).

### Systolic function

Bidimensional and line scan mono-dimensional (B and M mode, respectively) echocardiography screening allows assessment of LV chamber dimension and systolic function. Short axis projection is commonly used to measure LV dimension of interventricular septum (IVS) and posterior wall (PW), which will give information about LV hypertrophy, while LV diastolic dimension (LVD) and LV systolic dimension (LVS) will indicate changes in dimension of LV chambers (increased in dilatative cardiomyopathy).

Heart rate (HR) can be obtained from inputs coming from ECG and breathing heating pad integrated device that provide real time electrocardiogram information. Other parameters of LV are calculated using the following equation:

- FS% fractional shortening: (LVD-LVS)/LVD *100
- EDV end diastolic volume: LVD^3^ (D^3^ formula) or 7*LVD^3^/(2.4+ LVD) (Teichholz formula)
- ESV end systolic volume: LVS^3^ (D^3^ formula) or 7*LVS^3^/(2.4+ LVS) (Teichholz formula)
- EF% ejection fraction: (EDV-ESV)/EDV * 100
- SV Stroke volume: EDV-ESV
- CO cardiac output: SV * HR heart rate

**Figure 1:**
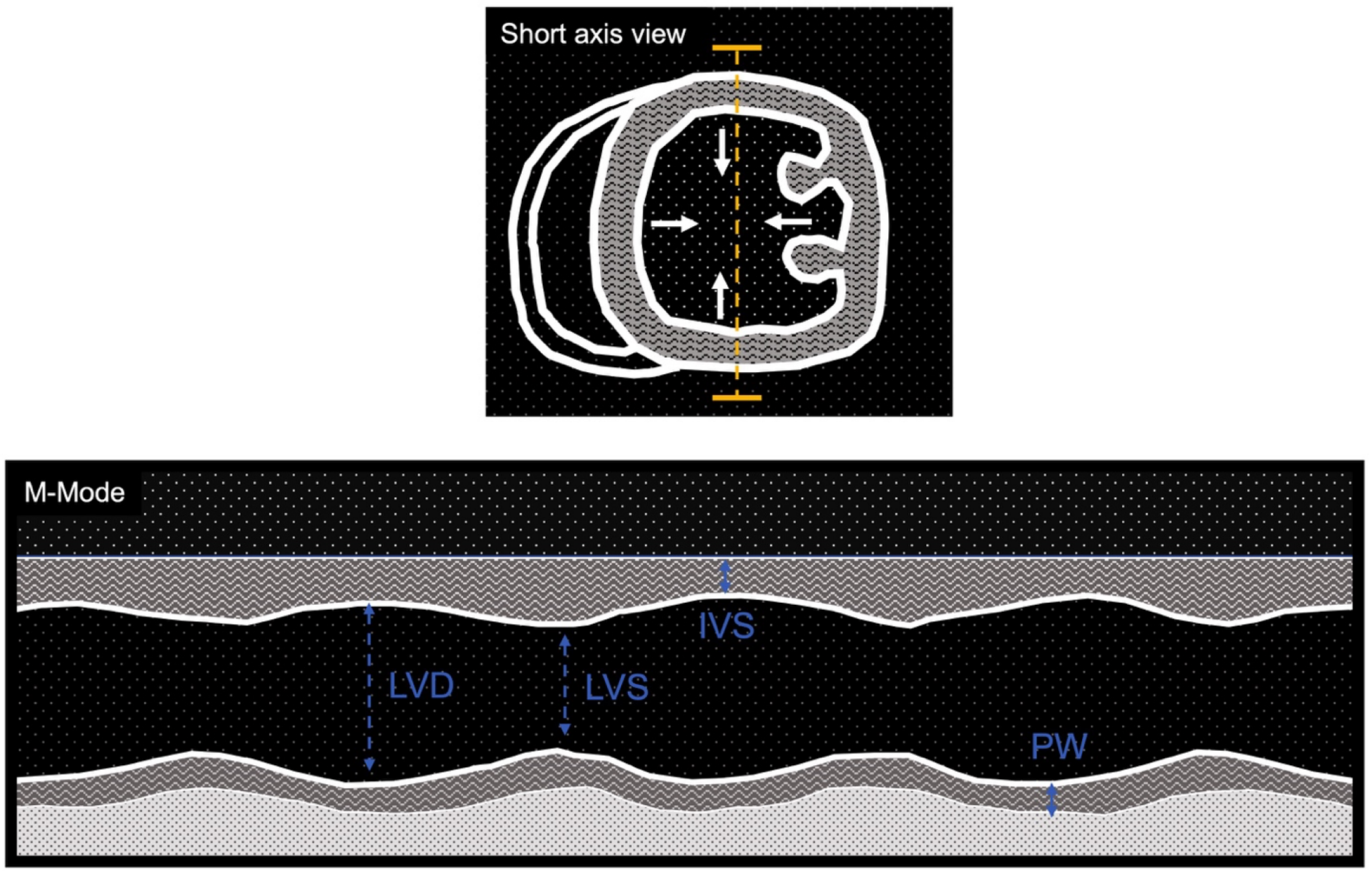
Cartoon depicting M-mode imaging of left ventricle short axis view. Top: short axis view of left ventricle (LV), which motions (arrows) are traced over time through a line scan (yellow dashed line). Bottom: mono-dimensional view of LV while is beating. Examples of measurements of left ventricle diastole (LVD), left ventricle systole (LVS), interventricular septum (IVS) and posterior wall (PW) dimensions.

Example of measures obtained with M-mode in short axis view of LV is shown in Figure 1, while a list of reference parameters for systolic parameters in healthy and diseased animal models are listed in Table 2.

**Table 2:** Echo cardio reference parameters in mice for systolic function and dimension of LV.

	Healthy	HFrEF	HFpEF	References
LVD (mm)	3.6–4.2	↑ ↑	←→/↓	(11, 16) (29)
LVS (mm)	1.5–1.9	↑ ↑ ↑ (MI) ↑ ↑ (TAC) ↑ (other DCM)	←→/↑	(16) (11)
FS%	40–55	↓ ↓ ↓ (MI) ↓ ↓ (TAC) ↓ (other DCM)	←→	(10, 11) (12–15, 30)
IVS (mm)	0.7–1.2	↑/←→ (MI) ↑ ↑ (TAC) ←→/↓ (other DCM)	↑	(18)
PW (mm)	0.6–1.2	↑	↑	(18)
HR (bpm)	450–600	←→	←→/↓	(10)

Increase (↑ up arrow), decrease (↓ down arrow), or no change (←→ horizontal arrows). LVD, left ventricle diastole; LVS, left ventricle systole; IVS, interventricular septum; PW, posterior wall; HR, heart rate. Reference values obtained from studies published that have used healthy mice and mice with HF.

### Diastolic function

**Figure 2:**
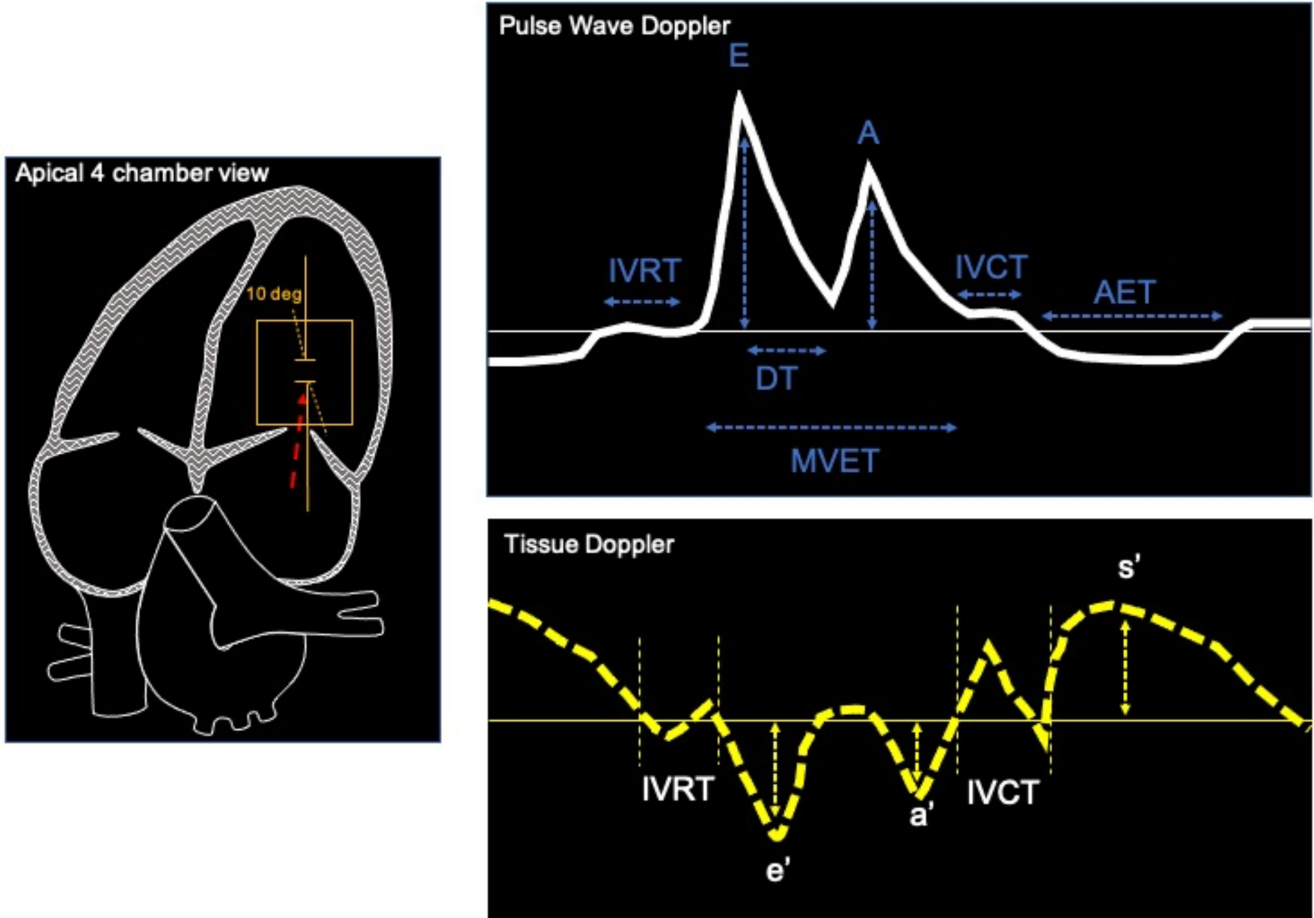
Example of apical 4 chamber view of the heart (left) used to record pulse wave Doppler (top) and tissue Doppler (bottom). Typical measurements of mitral blood flow with PW Doppler are: interventricular relaxation time, early and late mitral velocities (E and A waves), E wave deceleration time (DT), isovolumic contraction time (IVCT), aortic ejection time (AET). Tissue Doppler imaging are used to measure peak systolic velocities (s’), Peak early diastolic mitral annular velocities (e’) during early filling at septal or lateral corner of mitral annulus, Peak velocity in atrial systole (a’).

Non-invasive evaluation of LV diastolic function is mostly assessed through ultrasound imaging. Different echocardiographic parameters provide information about different aspects of diastolic function, its consequences and/or determinants. As in humans, Pulsed-Wave (PW) Doppler can be used in rodents to measure transvalvular flow-velocity profiles, which are particularly useful in assessing LV filling velocity, determined by the ratio between early (E) and late (A) diastolic trans-mitral Doppler flow velocities (E/A) and mitral E wave deceleration time (DT) (Figure 2, PW doppler and Table 3 reference values). PW-Doppler provides also information about the kinetics of LV systole, such as ejection time (ET) and isovolumic contraction time (IVCT), or LV diastole such as isovolumetric relaxation time (IVRT). The E/A ratio and the E-wave deceleration time (DT) are commonly used in human echocardiography to asses diastolic function of the heart. Prolongation of the isovolumic relaxation time (IVRT) or the mitral Doppler inflow E-wave deceleration time (DT) might reflect an impairment of left ventricle relaxation (32, 33). The fusion of the E- and A-wave is an indicator of diastolic dysfunction, however it is very challenging to measure these values consistently in mice since their high heart rates (>450 bpm) can cause artefactual fusion of E- and A-waves. Deeper anaesthesia slows down the heart rate in order to obtain a consistent measurement of E/A ratio in the mouse heart. However, this is undesirable due to the likelihood of associated cardiac depression. In fact, recent guidelines recommend to maintain the HR at physiological levels (>450 bpm) in order to avoid any cardio-depression effect and have reliable and reproducible data (22). Therefore, the assessment of these parameters through ultrasound imaging is unlikely to be useful in mouse echocardiography without a skilful and expert operator.

**Table 3:** Echo cardio reference parameters for diastolic function obtained through PW and TD Doppler.

	Healthy	HFrEF	HFpEF	References
E/A	1.1–1.5	↑	↑	(19, 20) (30, 31)
DT (ms)	16–31	↓	↓	(19, 20)
IVRT (ms)	16–19	↓	↓	(19, 20) (32, 33)
E/e’	19–38	↑	↑	(19, 20) (30, 32, 33)
LA area (mm^2^)	0.4–2.5	↑	↑	(31–33)

Increase (↑ up arrow), decrease (↓ down arrow), or no change (←→ horizontal arrows). (E and A waves) Early and late mitral velocities, (DT) E wave deceleration time, (IVRT) Isovolumic relaxation time; (E/e′) ratio, mitral inflow E wave/tissue Doppler mitral annulus velocity; (LA) left atrium. Reference values obtained from studies published that have used healthy mice and mice with HF.

The ratio between early and late filling (E/A ratio) is usually referred to the ventricular filling pattern on trans-mitral Doppler. It is important to consider that E/A ratio is influenced not only by myocardial relaxation and ventricular stiffness but also by the left atrial pressure (34). Moreover, the left atrial pressure is itself affected by left ventricular properties as it rises when the ventricle becomes stiffer, therefore when left ventricle relaxation is impaired, the E/A ratio decreases. Another issue is the “pseudo-normalization” of the E/A ratio that rises again with the progression of diastolic dysfunction due to the increase of left atrial pressure, which makes even more complex the interpretation of the data. Therefore, additional Doppler pulmonary venous flow evaluation is required to validate LV diastolic dysfunction (35).

Further increase in LV passive stiffness leads to a predominantly early filling pattern with abrupt termination of filling, which is defined as a “restrictive” pattern. This causes a marked decrease in IVRT and DT along with an increase in E/A ratio. Therefore, multiple parameters need to be measured for an accurate interpretation of cardiac physiology. Left atrial pressure is a very important hallmark to assess diastolic function, therefore parameters that provide information about left atrial properties are very useful. B-mode four chambers view is commonly used to measure the area of left atrium (LA), which increases when the pressure in left atrium is high.

Tissue Doppler imaging is used to integrate data obtained from PW Doppler imaging and assess global and regional cardiac function. Tracing the motion velocity of the mitral annulus enables the measurement of e’ (peak early diastolic mitral annular velocity), a’ (peak velocity in atrial systole), and s’ (peak systolic velocity) waves (Figure 2, Tissue Doppler trace). One drawback of the Tissue Doppler imaging method is that the analysis of radial function is limited to the anterior and posterior walls, while measurement of the circumferential function is limited to the septal and lateral walls, because Tissue Doppler can only measure velocities parallel to the ultrasound beam. Tissue Doppler imaging of mitral annulus provides another important parameter, the E/e’ ratio (mitral inflow E wave/Tissue Doppler mitral annulus velocity), which correlates with left atrial pressure, while the size of left atrium provides a useful indication of chronically elevated left atrial pressure (36). PW and Tissue Doppler echocardiography are performed using a four chambers view, pointing the caliper (less than 20 degrees angle) at medial mitral annulus (Figure 2, four chamber view) to obtain the following parameters:

Parameters obtained with PW Doppler:

- Early and late mitral velocities (E and A waves)
- E wave deceleration time (DT)
- Isovolumic relaxation time (IVRT)
- Isovolumic contraction time (IVCT)

Parameters obtained with Tissue Doppler:

- Peak systolic velocities (s’)
- Peak early diastolic mitral annular velocities (e’) during early filling at septal or lateral corner of mitral annulus.
- Peak velocity in atrial systole (a’)

## Magnetic resonance imaging

Magnetic resonance Imaging (MRI) is another non-invasive though expensive procedure that has been employed to establish imaging parameters that can be adopted into clinical practice to predict cardiovascular outcomes also in animal models of cardiomyopathy. This method provides high-quality resolution in static organs although, the quality of cardiac dynamic imaging might be challenged by the small size, the high heart rate and signal noise from respiration. Simultaneous ECG and respiration recording is commonly performed during MRI procedures for gating and synchronization of imaging with cardiac and respiratory cycles. Major limitations with the use of MRI include the high cost of the instrument and contrast agents, the massive size of the equipment, time and resource-intensive protocols, lower temporal resolution, signal-to-noise ratio limitations, which limit its more extensive use in preclinical studies. The major advantage of this technique is the accuracy in identifying tissue characteristics that correlate with histopathological findings, which makes this non-invasive procedure unique and very useful prognostic tool. In particular MRI allows detection of moderate to severe diffuse fibrosis, which makes MRI the only methodology available for *in vivo* assessment of cardiac fibrosis. This could enable studies that test new therapeutic strategies that can prevent, delay, or even reverse the effects of cardiac remodelling. Moreover, conventional two-dimensional cine images allow more accurate measurements of LV volumes and mass as the endocardium and epicardial areas calculations are performed in multi-planar short and long axis to cover the whole LV. The following parameters are commonly used to assess cardiac function and anatomy through MRI:

- LVEDV, left ventricle end diastolic volume: Σ (endocardial area over all slices at diastole) * thickness of the slice
- LVESV, left ventricle end systolic volume: Σ (endocardial area over all slices at systole) * thickness of the slice
- SV, stroke volume: LVEDV-LVESV
- EF (%), ejection fraction: SV/LVEDV * 100%
- CO, cardiac output: SV * HR
- RS, radial shortening: short axis (endocardial diameter at diastole − endocardial diameter at systole)/endocardial diameter at diastole * 100%
- LS, longitudinal shortening: long axis (endocardial diameter at diastole − endocardial diameter at systole)/endocardial diameter at diastole * 100%
- LVWV, left ventricle wall volume: Σ (epicardial area over all slices) * thickness of the slice − Σ (endocardial area over all slices) * thickness of the slice
- LVM, left ventricle mass: LVWV * 1.05 (myocardial density = 1.05 g/ml)

Quantitative evaluation of cardiovascular function with MRI imaging must consider the big gap in spatial resolution that exists between rodents (5- and 10-fold higher) and humans as the human heart size is 1,000-fold bigger with 5–10 times slower heart rate. The higher spatial resolution results in losing 100–1,000% of inherent signal/noise ratio (SNR). The signal-to-noise ratio and the image resolution is determined by the range of magnetic field strengths of the MRI scanners, which ranges between 4.7 and 9.4 T (for small animals) and from 1.5 to 3.0 T (for large animals). However, clinical 1.5 and 3.0 T scanners can be adapted for MRI imaging in small animals as described by Gilson and Kraitchamn (37), since high-field systems are not readily available at many institutions.

To ensure reproducibility and reliability of cardiovascular imaging studies, investigators should consistently report type and dose of anaesthesia, duration of the imaging procedure, and monitored heart rate and body temperature, which decrease while under anaesthesia during image acquisition. Temperature monitoring can be done using an MR-compatible rectal probe. Temperature maintenance can be accomplished by integrating a low-flow water-based heating blanket or pad into the cradle or by using heater fan over the animal.

It is possible to assess diastolic function using a temporal resolution of 1 msec using high-temporal-resolution cinematic magnetic resonance imaging (CINE MRI) (38), although this procedure is user-dependent and it is not commonly used in preclinical practice. Further methodological details of MRI procedures can be found in other excellent articles (39, 40).

## PV loop analysis through invasive catheterization of left ventricle

### History of PV loop analysis

Invasive left ventricle catheterization to measure pressure and volume has been introduced in dog's hearts in the 70′ using *ex vivo* settings (41) and later in the 90′ by Kass and colleagues in humans (42, 43). Microminiaturization of conductance catheters enabled the use of PV loop analysis for preclinical studies *in vivo* (44). This technique is the most rigorous and comprehensive way able to provide extensive information of systolic and diastolic function either dependent or independent by LV load and it is still considered the “gold standard” method to study cardiac pathophysiology in preclinical settings. Despite its invasiveness, this sophisticated methodology can be used to characterize cardiovascular function in transgenic mice, to test the hemodynamic effects of pharmacotherapies and studying cardiovascular pathophysiological conditions using small animals for genetic and pharmacological investigations (45). The main and unique advantage of this method compared to other non-invasive procedures (e.g., echocardiography, MRI) is that PV loop analysis can provide measures of LV performance independently of vascular loading conditions and heart rate.

Detailed protocols of PV loop analysis procedures have been reviewed in other excellent articles (45–48). The aim of this article is to overview the methodology for assessing systolic and diastolic dysfunction in mice including calibration of the equipment, surgical procedures and data interpretation.

### PV loop description

**Figure 3:**
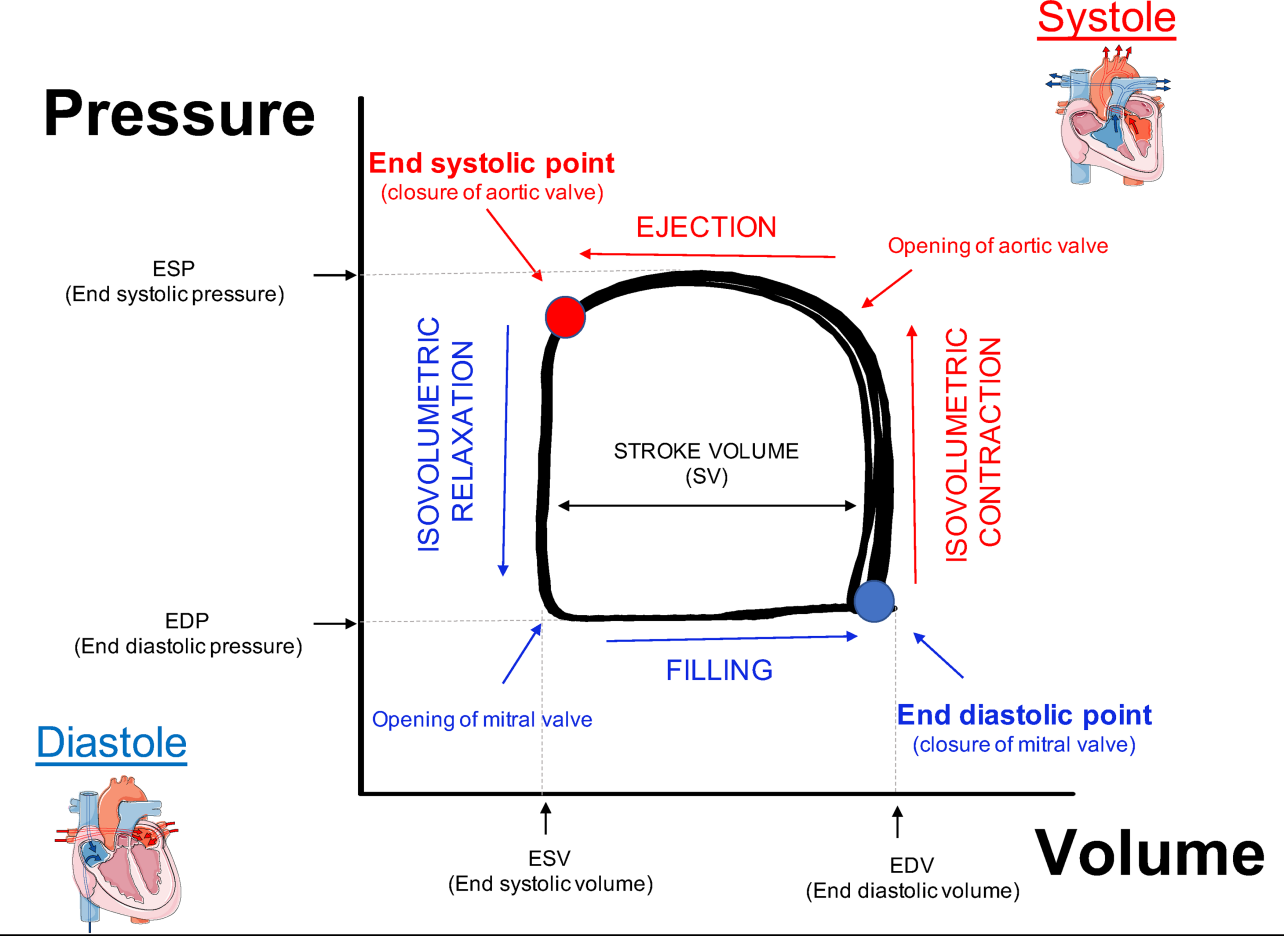
Description of PV loop. End diastolic point (blue circle): the LV has completed the blood filling phase and the mitral valve is closed. Isovolumetric contraction: aortic and mitral valves are closed while the ventricle starts to contract causing an increase of pressure without changes in volume. Ejection phase begins when the aortic valve opens. The end systolic point (red circle): when the aortic valve closes and the relaxation phase begins to relax. Isovolumetric relaxation: all the valves are closed during ventricle relaxation, which leads to reduction of pressure without altering the volume. Filling phase: the LV is filled with blood following opening of the mitral valve.

Plotting hemodynamic changes of LV pressure on Y axis and volume on X axis will give a rectangular shaped loop known as PV loop. An easy way to understand the cardiac cycle, which is very helpful for educational purpose, is to draw a line from the top left corner to the bottom right corner of the loop in order to reveal two triangles where the one on the top right refers to the LV systole, while the triangle on the bottom left represent the LV diastole. To better interpret and understand the PV loop data we will describe how pressure and volume change during cardiac cycle starting from the end of LV diastole (Figure 3, PV loop description). The point on the bottom right corner (blue circle) represents the end diastolic point. The LV has completed the blood filling phase and starts to contract causing indirectly the closure of mitral valve which leads to an increase of pressure without changes in volume (Isovolumetric contraction). When the pressure in the ventricle exceeds the pressure in the aorta, the aortic valve opens (top right corner) and the ejection phase begins. This allows the LV to reach its peak in pressure during systole. The relaxation phase starts at the end systolic point (top left corner, red circle) when the aortic valve closes as the LV pressure is now lower than aortic pressure. As the ventricle begins to relax with all valves closed there is a reduction of pressure without altering the volume (Isovolumetric relaxation). The LV will start the blood filling phase when the pressure in the LV is lower than the pressure in the atrium, which causes the mitral valve to open. In human beating hearts it has been demonstrated that the very rapid initial filling phase depends on the elastic properties of the heart. Indeed, in healthy condition the compression of the elastic element of the ventricle together with three-dimensional twisting deformation of the myocardium during systole, generate restoring forces resulting in a rapid negative diastolic pressure upon relaxation, while diseased and stiffed hearts manifest blunted protodiastolic suction (49, 50). The cardiac cycle concludes at the end of the filling phase (end diastolic point), when the mitral valve closes.

The peak of maximal pressure and minimal pressure are transposed for each cardiac cycle on *Y* axis to obtain values of end systolic and end diastolic pressure (ESP, EDP respectively). The difference between end diastolic volume and end systolic volume (transposed on *X* axis) defines the stroke volume of LV, which will be used to calculate cardiac ejection fraction.

End systolic point and end diastolic point are important to measure load independent cardiac function, e.g., the end systolic and end diastolic pressure volume relationship (ESPVR, EDPVR) obtained upon reduction in ventricle preload (described below in preload reduction paragraph).

### Surgical procedures for LV catheterization

Understanding the principle of this method involves two parts: (a) master the surgical procedure for placement the catheter in LV for pressure and volume measurements, (b) analyse the data obtained. For surgical procedures, isoflurane (1%–2%) has become the most popular anaesthetic for PV loop and other for non-invasive cardiac physiology analysis in mice (51), while the mixture of Ketamine and Xylazine has been shown to be unsuitable for physiological measurement mostly for the cardio depressant effect of Xylazine, which reduce HR and LV function compared to isoflurane. The use of different types of anaesthesia used in PV loop analysis contributes to the large interlaboratory variability of data for hemodynamic parameters (Table 4, hemodynamic parameters reference values in healthy mice) (46, 48).

**Table 4:** Selected hemodynamic parameters in healthy anaesthetised mice and their change in pathologic conditions.

	Healthy	HFrEF	HFpEF	References
HR (bpm)	470–640	←→	←→	(46, 48) (32, 33)
ESP (mm Hg)	92–118	↑ ↑ (in TAC) ←→/↓ (in MI and other DCM)	↑	(46) (19, 20)
EDP (mm Hg)	1–6	↑	↑↑	(46) (19, 20)
ESV (µl)	7–21	↑ (in TAC) ↑↑ (in MI and other DCM)	↑/←→/↓	(46) (52)
EDV (µl)	25–53	↑ (in TAC) ↑↑ (in MI and other DCM)	↑/←→/↓	(46) (52)
SV (µl)	17–30	↓ ↓	←→/↓	(46)
CO (ml/min)	8–16	↓ ↓	←→/↓	(46) (45, 51, 53)
EF %	55–72	↓	←→	(52) (32, 33)
Ea (mmHg/µl)	3–7	↑	↑	(52) (19, 20)
dP/dt_max_ (mmHg/s)	8,200–14,200	↓	←→/↓	(46, 48)
ESPVR slope, E_es_ or E_max_ (mmHg/µl)	7–14	↓ ↓	←→	(54)
PRSW (mmHg)	58–99	↓ ↓	←→/↓	(46, 48)
dP/dt_max_/EDV (mmHg/s/µl)	580–799	↓ ↓	←→/↓	(46, 48)
−dP/dt_min_ (mmHg/s)	(−)6,700–10,500	↓	↓	(46, 48) (32, 33)
Tau (ms)	7–12	↑ ↑	↑ ↑	(54) (32, 33)
EDPVR slope, β (mmHg/µl)	0.04–0.12	↑ ↑	↑↑	(19, 29, 55, 56)

HR, heart rate; ESP, end systolic pressure; EDP, end diastolic pressure; ESV, end systolic volume; EDV, end diastolic volume; SV, stroke volume; CO, cardiac output; Ea, arterial elastance (index of ventricular afterload); EF, ejection fraction; dP/dt_max_, peak rate of pressure rise; E_es_ (E_max_), end systolic elastance (slope of end-systolic relationship); PRSW, preload-recruitable stroke work (slope of stroke work-EDV relationship); (dP/dtmax)/EDV, slope of relationship between dP/dtmax and EDV; −dP/dt_min_, Peak rate of pressure decline; Tau, relaxation time constant; EDPVR, β, slope of end diastolic pressure volume relationship (more steep, increased stiffness). Reference values obtained from studies published that have used healthy mice and mice with HF.

### Anaesthesia with isoflurane

The mouse is sedated in the induction chamber saturated with 2%–3% isoflurane. Once the mouse is asleep the sedation is maintained through a mask connected to a respirator providing anaesthesia mixture (1.5%–2% isoflurane). The hair from the neck and the chest are removed using depilatory cream while the skin is cleaned with water and prepared for surgery with 0.5% chlorhexidine. Then a mid-sternal incision of the skin from the neck to the sternum will expose the salivary glands. These will be then separated in order to expose the trachea and confirm the correct placement of the endotracheal cannula. The endotracheal cannula can be custom made using an 18 G blunted needle. Before inserting the cannula, the mask is removed and the tongue is gently pulled to avoid obstruction during cannula insertion. Finally, the cannula is connected to a respirator providing gas anaesthesia mixture. For mice surgery, the respirator should be set at a stroke rate of 120–140 breaths per minute and a stroke volume (tidal volume) of 0.2–0.4 mm.

### Cannulation of left jugular vein for saline infusion

The left jugular vein is the most common access point used to infuse fluids during PV loop analysis. The catheter can be custom made by using a PE-20 tube connected to a 29 G needle. Note that the needle will be inserted into the vein with bevel of the needle facing up, while the PE-20 tube will serve as connection to the adapter switch connected to other syringes containing different solutions. Before placing the catheter in the jugular vein, it is recommended to flush the catheter with the first solution that needs to be infused to check any possible obstruction of the catheter of the cannula. After exposing the jugular vein by moving proximally the salivary gland, the left jugular vein can be cannulated. In order to infuse different solutions in the same animal the cannulation tube is connected to a switch adapter so that more syringes (e.g., containing saline, hypertonic solution or drug that needs to be tested) can be connected to the same cannula and inject different solutions. Upon jugular vein cannulation, 12.5% albumin in normal saline is infused at 5 μl/min, after an initial 50-μl bolus to counteract the peripheral vasodilatation and hypotension induced by anaesthesia. PV loop analysis can be employed to assess any acute effects on cardiac physiology that a wide variety of drugs may induce upon infusion.

### LV catheterization

The catheter most commonly used for PV loop analysis in mice is the Millar SPR 839 (size 1.4 French), which contains 4 electrodes with pressure sensor centred between E2 and E3 (45). Before starting any experiment, the catheter needs to be calibrated for volume and pressure according to manufacturer's instruction.

Before catheterization, the catheter tip needs to be pre-soaked in warm saline (37°C) for 30 min (at least) before use. This can be done by inserting the tip through a 1-ml syringe containing physiological saline solution placed on a heating pad.

There are two ways of inserting the catheter in left ventricle of mice: the closed chest approach from the right carotid, or the open chest approach directly from LV apex.

### Closed chest approach

**Figure 4:**
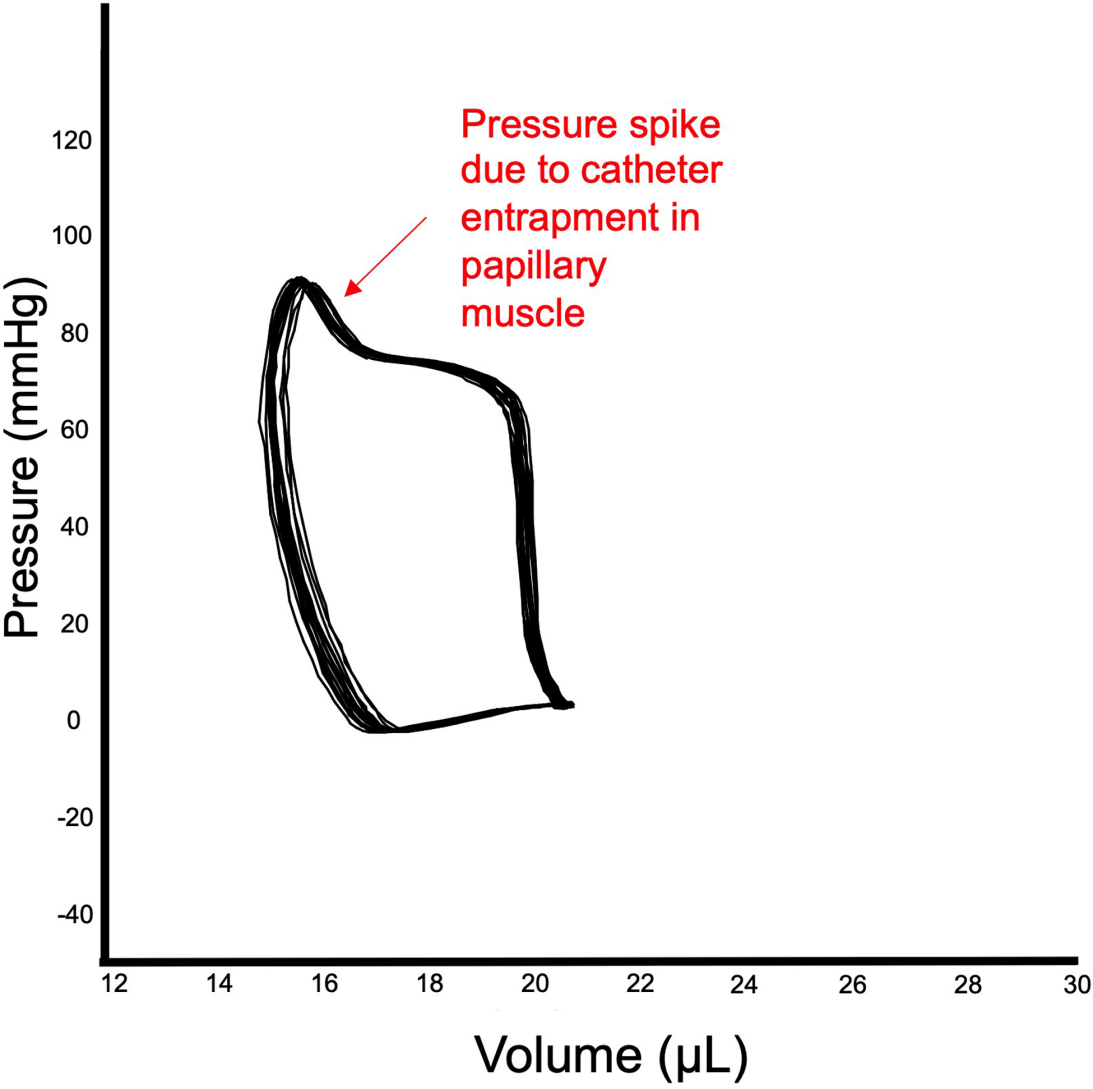
Catheter entrapment. Example of entrapment of catheter in papillary muscle of LV. The end of the systole reveals a spike in LV pressure, while stroke volume is relatively small.

The closed approach consists in inserting the catheter through the right carotid, which will retrogradely reach the LV chamber. The critical part of this approach is to overcome the loop of aortic arch before reaching the LV, which is quite challenging due to the small size of the vessels and the limitation to adjust the catheter orientation within the ventricle. A potential problem with retrograde insertion of the catheter is the risk of outflow tract obstruction crossing the aorta, which becomes a significant issue in smaller hearts. The diameter of the mouse aorta varies from 0.8 to 1.2 mm while commercially available PV catheters have a diameter about 1/3 of mouse aorta diameter (ranging from 0.33 to 0.47 mm, 1.0 to 1.4 French respectively) (48). The closed-chest approach is more suitable for more prolonged experiments, because normal intrathoracic pressures are maintained, there is less risk of bleeding and the animals are more stable for a longer period of time. In a chronic heart failure model induced by ligation of the left anterior coronary artery characterized by the scar formation in the apex area the carotid approach should also be used. However, catheter entrapment is a common artifact of measurement that can be generated by direct compression of the transducer by a papillary muscle or other dynamic structure within the ventricle. This artifact can be detected by the typical spike in pressure at the end of systole, which often results in very small changes in LV volume during cardiac cycle (see representative image Figure 4). This can impact the analysis of PV loop data, because most methods for determining systolic function use the maximum pressure derivative. Therefore, data sets need to be examined closely for catheter entrapment to ensure meaningful data.

### Open chest approach

This protocol is commonly used for drug testing and it consists in inserting the catheter in the LV via the apex, therefore it is easier to confirm the proper placement of the catheter in the LV, and the PV loop recording is more consistent while the whole procedure can be executed in less time. The open chest approach is also indicated if the carotid artery is severely atherosclerotic (e.g. in ApoE mice fed with a high-fat diet), or when the aortic valve is calcified (e.g., in advanced aging models) or in transverse aortic constriction (TAC)-induced hypertrophy and heart failure models. However, this procedure requires to cut the diaphragm in order to expose the apex of LV, which leads to loss of thoracic pressure impacting the venous return. Therefore, it is important to use consistently one surgical approach in order to obtain reliable PV loop data.

### Total peripheral resistance (TPR)

This parameter is very important for new drug that may also affect peripheral resistance. Using the carotid approach to insert the catheter provides the advantage of easily recording arterial pressure from the carotid artery at the start or at the end of the experiment, therefore total peripheral resistance (TPR) can be calculated afterwards as follows: *TPR = (mean arterial pressure–mean venous pressure)/cardiac output*.

Alternatively with the open chest approach, one of the femoral arteries can be cannulated with a PE tube (P10) and connected to another pressure transducer recording on separate record channel. This allows to calculate the total peripheral resistance (TPR) changes throughout the experiment.

### Protocol for open chest approach

This is a quick and reproducible approach to insert the catheter in LV. In anesthetized and intubated animals connected to a mechanical respirator, the xiphoid process is exposed separating the skin from the ribs cage using blunt forceps. To reduce the risk of bleeding the operator should avoid to cut the ribs (particularly if a heat cauterization unit is not available). It is recommended that the operator lifts up the xiphoid process of the mouse and cut the abdominal muscles underneath the ribs to expose the diaphragm. This latter can then be easily cut along the junction of the ribs cage. This will prevent any major bleeding in order to access the apex of the heart.

In order to have a clear view of the heart orientation, the ribs can be lifted up using chest retractors. The pericardium is gently removed from the heart with forceps.

The apex of the heart is stabbed wound with a 25 G needle attached to a syringe to prevent any sudden drop in ventricle pressure following the stabbing.

The orientation of the needle represents a critical point in order to have a clear noise-free PV loop signal. In fact, the needle needs to enter into left ventricle through the apex no more than 2–4 mm deep maintaining the orientation of the needle toward the base of the heart. Then the catheter tip can be quickly inserted into the left ventricle using the access point created by the needle. The catheter is pushed until the proximal electrode on the catheter (E4) is just inside the ventricular wall, while the orientation is parallel to the long axis of the ventricle. The correct position of the catheter can then be adjusted in order to obtain rectangular-shaped PV loops. Baseline PV loops are recorded after stabilization of the signal at steady state or following inferior vena cava occlusions in order to vary the preloads. This latter is particularly used to obtain various load-independent indices of systolic and diastolic function (see inferior vena cava occlusion below).

At the conclusion of the experiment, the catheter is gently pulled back through the stab wound and the animal is euthanized. The blood is collected in tubes with heparin and used for volume calibration with cuvettes (see volume calibration below).

It is recommended to place immediately the tip of the catheter into a syringe filled with saline solution (NaCl 0.9%) to prevent clotting. Finally, the catheter should be cleaned with detergent (e.g., Alconox) according to manufacturer's instructions as proper care of the catheter will considerably prolong its useful life.

### Preload reduction through inferior vena cava (IVC) occlusion

**Figure 5:**
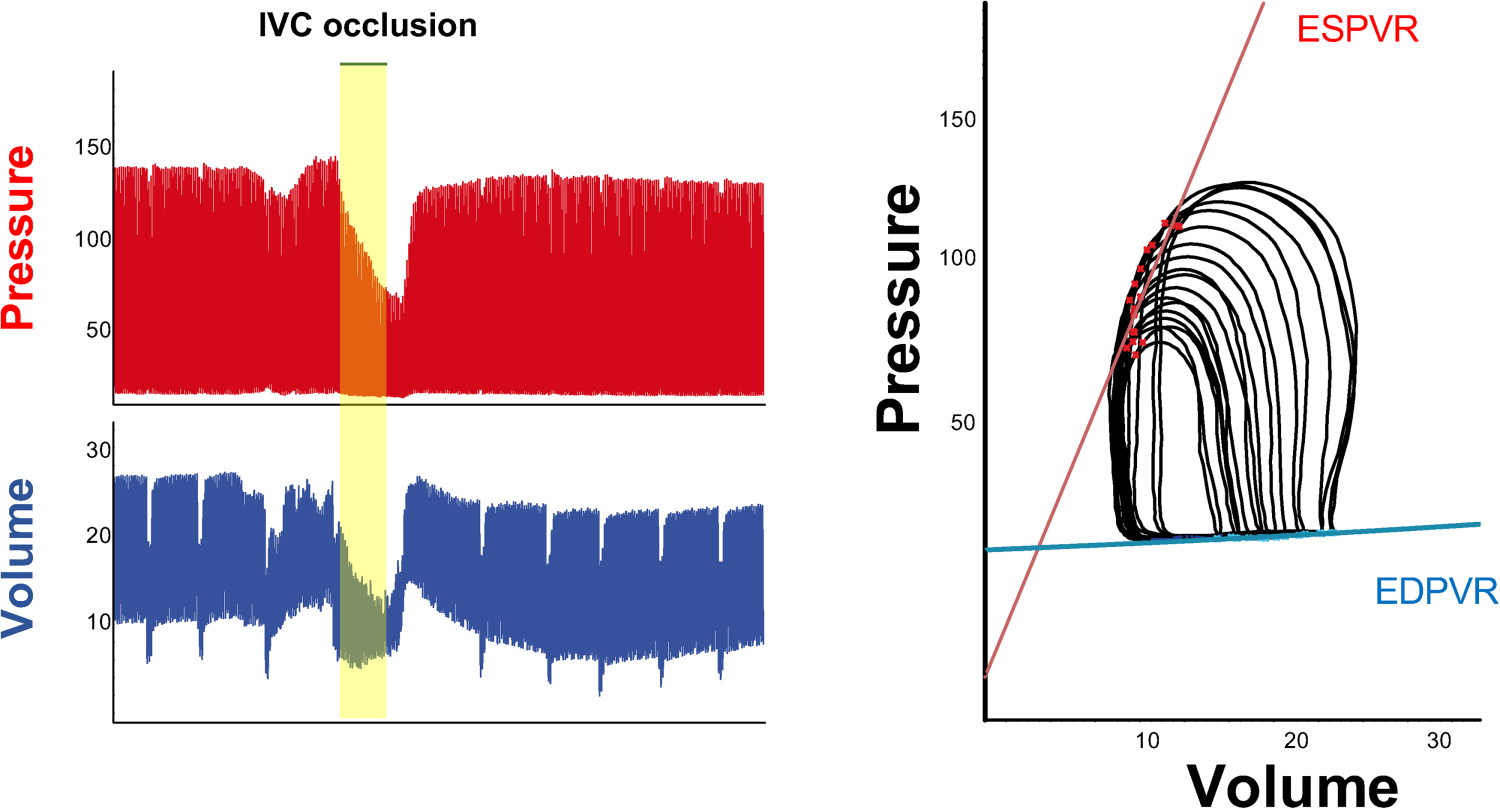
IVC occlusion. Example of change in pressure and volume following preload reduction through inferior vena cava occlusion (left). On the right, series of smaller PV loops shifting to the left generated over a range of decreasing ventricular preloads. Each of these loops has its own end systolic point and end diastolic point, which will be used to calculate load independent parameters such as ESPVR (red line) and EDPVR (blue line).

Inferior vena cava occlusions can be performed in open-chest respirated animals by lifting a suture placed around the vessel, or compressing the vessels with a blunted forceps. Load independent measures of contractility and relaxation are obtained by reducing LV preload through occlusion of IVC for few seconds. By reducing the preload, there will be a reduction of pressure and volume in the ventricle (Figure 5, on the left) and a decrease in the amount of stretch of the ventricle prior to a contraction. Occlusion of IVC will be displayed as a series of smaller PV loops shifting to the left generated over a range of decreasing ventricular preloads. Each of these loops have their own end systolic point and end diastolic point (red and blue points in loops in Figure 5) that will be used by the software to calculate end systolic and end diastolic pressure volume relationship (ESPVR, EDPVR) as load-independent parameters of ventricle contractility and relaxation, respectively.

### Load independent parameters

The interpretation of end systolic and diastolic pressure volume relationship (EDPVR and ESPVR) provides important information about the heart performance independently of load conditions. The maximal pressure that can be developed by the ventricle at any given LV volume is referred as end-systolic pressure volume relationship (ESPVR). This parameter is an improved index of systolic function over other hemodynamic parameters like ejection fraction, cardiac output, and stroke volume. The passive properties of the myocardium (passive filling curve for the ventricle) are described by the end-diastolic pressure volume relationship (EDPVR).

### Volume calibration

The volume raw data are generally recorded as relative volume units (RVU), which can be converted in microliters (μl) in two steps: conversion of RVU to μl with blood cuvettes and correction for the parallel conductance volume with saline calibration. It is recommended to perform blood cuvette calibration and saline calibration for each animal at the end of each PV loop recording in order to have an accurate measurement of volumes. However, due to the limited amount of blood that can be collected from each mouse, it is also possible to pool together blood from different animals and perform one blood cuvette calibration at the end of each experimental day.

#### Step 1: convert the RVU in µl using blood cuvettes

Blood cuvettes are mock-up cylinders with known volumes that are filled with heparinized warm blood (body temperature) placed on the heating pad. The cuvettes are filled with blood using 22 G blunted needle long enough to reach the bottom of the cylinder (length 3.9 cm) avoiding formation of air bubbles. The catheter is inserted in each cuvette, submerging the 4 electrodes and keeping the position steady for 10–20 s, to record the changes in conductance values RVU. A calibration curve will be generated using 4–5 known cuvettes which will enable the conversion of the data from RVUs into units of true volume (μl) using PV loop analysis software. However, the conductivity of the heart muscle that surrounds the LV blood pool (parallel conductance) makes these estimated volume signals still larger than expected. Therefore, intravenous hypertonic saline calibration is performed (described below), to account and correct for parallel conductance.

#### Step 2: hypertonic saline calibration

Hypertonic saline calibration is performed because data generated from conductance catheters depend on the relationship between volume and conductance. Changes in conductance are measured by changes in current flowing from proximal to distal electrode, which is mostly given by movement of blood pool. However, the contribution of the ventricular wall, termed parallel conductance, must be subtracted to obtain absolute LV volume measurements (47). Indeed, the current applied to the excitation electrodes on the catheter does not go only through the blood, but some of the applied current flows also into the surrounding muscle, which is a conductor rather than an insulator, often causing an overestimation of the blood volume within the ventricle. Therefore, the heart muscle acts as a shunt to the applied current, referred as parallel conductance, or in volume calculations as parallel volume (Vp). To obtain a value for Vp, a bolus 15–20 μl of hypertonic saline (NaCl 30%) is injected through jugular vein into the animal at the conclusion of the experiment. This will cause a visible shift to the right in PV loops (increase volume conductance signal) without significant decrease in the pressure signal amplitude. The parallel volume (Vp) is calculated by solving a system of linear equations to locate the intersection of two lines. The first line is represented by the saline calibration data plotting end diastolic volume vs. end systolic volume (ESV) for each cardiac cycle during the phase where the volume signal appears to rise following the hypertonic saline bolus. The other line derives from the equation EDV = ESV, which represents equal end-systolic and end-diastolic volumes or in other words as the equivalent of a heart chamber devoid of blood. Then the PV loop software calculate the parallel volume (Vp) of muscle tissue that corresponds to the value at the intersection of the EDV = ESV line and the saline calibration line. Further details of the method and its underlying theory have been reported previously (45). It is recommended to perform at least 2–3 saline calibrations in each animal to minimize possible variability. The calculated Vp value from saline injection together with the parameters derived from blood cuvette calibrations will be used by the PV loop software (e.g., Lab Chart 8, Ad Instrument) to calculate the true volumes in microliters.

### Alpha calibration (optional)

It is possible to use other non-invasive procedures (i.e., echocardiography or MRI) to measure of LV volumes in order to validate volume calibration performed with catheter (Alpha correction) (45). This represents an optional step where the alpha coefficient of Baan's equation is calculated by dividing the cardiac output (CO) or stroke volume (SV) recorded by PV loop module by the CO or SV calculated by other non-invasive methodologies. However, from a practical point of view the calibration can be performed independently of other non-invasive methodologies.

### Duration of PV loop procedure

The whole procedure can last from 30 to 120 min depending on time necessary for anaesthesia, surgery and calibrations for one animal, however the experience and surgical skills of the investigator and study design play a critical role in the proper execution of this complex procedure. Starting with a few control animals on the day of the experiment will ensure that everything is optimized and working well before proceeding with measurements in pathological states or following pharmacological treatment. Indeed, this could be useful to minimize potential errors in case the catheter needs to be replaced in the middle of the study by another one with slightly different properties.

## Interpretation of hemodynamic parameters obtained from PV loop analysis

This sophisticated methodology enables simultaneous measurements of both pressure (on *Y* axis) and volume (on *X* axis) data in murine ventricle for each cardiac cycle both at steady state and during preload changes (IVC occlusion). Therefore, several hemodynamic parameters of systolic and diastolic function can be derived from the analysis of pressure and volume relationship. Generally, 10–12 cardiac cycles are selected and analysed with PV loop software to obtain the hemodynamic tables including systolic and diastolic parameters. We will describe the parameters that are most commonly used in cardiovascular preclinical studies and how they are calculated by the software.

The heart rate, expressed as beat per minutes (bpm), is calculated by the software by either automatically counting the cardiac cycles over time or by analysing the ECG signal recorded on a separate channel of the PV loop analysis software.

End systolic pressure (ESP), end diastolic pressure (EDP), expressed in mm of Hg, represent the maximal pressure and the minimal pressure respectively, that have been recorded during each cardiac cycle. End systolic volume (ESV) and end diastolic volume (EDV) are calculated following volume conversion in microliters (µl) (described above), indicate the volume of blood during ventricle contraction and relaxation respectively.

### Systolic parameters

Stroke volume (SV) indicates how much blood is pumped by the ventricle following each contraction and it is obtained by subtracting the end diastolic volume to the end systolic volume (SV = EDV—ESV) and it is expressed in µl.

Ejection fraction (EF) represents the fraction of end diastolic blood volume that is propelled by the ventricle during each contraction. It is calculated dividing the stroke volume to end diastolic volume (EF = SV/EDV). This parameter is expressed in percent.

Cardiac output (CO) represents the amount of blood that is propelled by the ventricle each minute. CO it is calculated by multiplying the stroke volume to the heart rate (SV * heart rate). The unit of measurement is µl/min.

The maximal rate of rise of left ventricle pressure (dP/dt_max_) is an index of contractility that relies on loading condition of the ventricle.

Arterial elastance (Ea) it is an index of arterial vascular load (LV afterload) and it is calculated by the ratio between end-systolic pressure and stroke volume (ESP/SV). The unit of measurement is mm Hg/µl.

Stroke work (SW) refers to the work done by the ventricle to eject a volume of blood. It refers to the area of the PV loop and is calculated by mean arterial pressure times cardiac output (MAP*CO).

### Load independent systolic parameters (obtained following preload decrease with IVC occlusion)

The dP/dt_max_ –end-diastolic volume relation provides a load-independent contractility index, as preload dependence of dP/dt_max_ is effectively reduced by using this regression.

End-systolic pressure volume relationship (ESPVR) describes the maximal pressure developed by the ventricle at any given LV volume and is a measure of cardiac contractility.

Maximal elastance (Ees or Emax) is equal to the slope of the ESPVR curve. It is an index for chamber end systolic elasticity/stiffness and can be a useful measure of contractile function, particularly to assess acute changes.

Preload recruitable stroke work (PRSW) is another index of load independent contractility, which is obtained by plotting stroke work vs. end-diastolic volume for the set of load-altered loops.

### Diastolic parameters

The minimum rate of pressure changes in ventricle (dP/dt_min_) is the minimum derivative of change in diastolic pressure over time. Left ventricle traces first derivatives dP/dT_max_ and dP/dT_min_ give an indication of systolic and diastolic function where a decrease indicates an impaired function.

Isovolumic relaxation time constant (TAU, *τ*) represents the exponential decay of ventricular pressure during isovolumetric relaxation (pre-load-independent parameter).

### Load independent diastolic parameters (obtained following preload decrease with IVC occlusion)

End diastolic pressure volume relationship (EDPVR) is a load-independent index of the passive filling properties of the ventricle and the passive properties of the myocardium. The slope of EDPVR is a measure of myocardial compliance (ventricle passive filling), which is the reverse of ventricular stiffness.

### Hemodynamic changes during heart failure

In diseased animals, the shape of the PV loops may not be rectangular. In models of LV pressure overload (i.e., TAC), the LV walls become thicker and LV chamber size decreases or remains unchanged (52). The increased wall thickness can reduce compliance of LV, therefore the slope of EDPVR increases. During compensatory hypertrophy the systolic pressure is not suppressed and the end-systolic pressure (*Y*-axis) is not changed.

**Figure 6:**
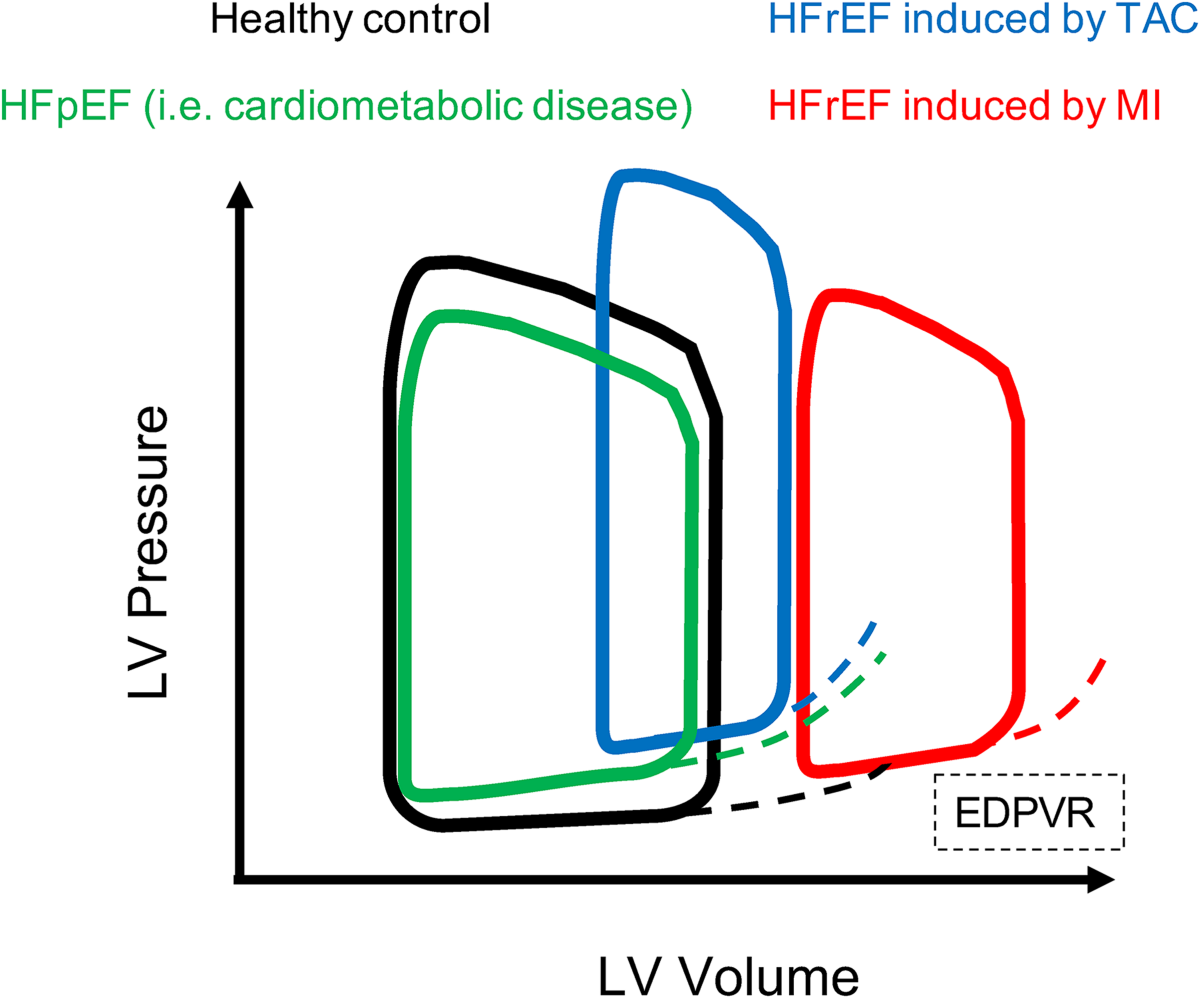
PV loops in healthy and HF models. Examples of PV loops. In black PV loop of healthy model, then models of HFrEF present PV loop shifted to the right. In particular HFrEF model induced by MI (red) presents enlargement of the LV chambers and impaired contractility, while HFrEF model induced by TAC (blue) presents increased ESP and EDP due to aggravated afterload, and increased EDPVR therefore the loop is shifted on the top right compared to healthy control. The model of HFpEF (green) is characterized by preserved contractile function, but impaired relaxation of hypertrophied and fibrotic LV therefore the PV loop is slightly shifted to the top and presents increased EDPVR compared to healthy control.

In models of HFrEF the EF is drastically reduced and there are clear signs of contractility disfunction (e.g., severe reduction of stroke volume and cardiac output), accompanied by diastolic disfunction (increased Tau and slope of EDPVR) (54). In models of HFpEF the EF is preserved or there might be mild signs of contractility dysfunction, however diastolic disfunctions manifest as decrease in dp/dt_min_, while Tau and slope of EDPVR increase (19, 29, 55, 56). In particular, an increased stiffness of the myocardium or a decreased compliance of LV will appear in PV loop analysis as an increased slope of EDPVR, which can be observed in both HFrEF or HFpEF models. Examples of PV loops in healthy and diseases models are depicted in Figure 6. In dilated cardiomyopathies (i.e., MI), ESV and EDV increase and LV pressure can remain unchanged, therefore the ESPVR and EDPVR are shifted to the right. Alternatively, when end-systolic pressure decreases and end-diastolic pressure increases the PV loop appears smaller (shorter) and rightward shifted (45, 51, 53). Upward shift in the EDPVR is observed in dilated cardiomyopathy associated with fibrosis and diastolic dysfunction. Chronic changes in Ees from heart disease can be a consequence of cardiac remodelling such as hypertrophy and fibrosis, and thus is not simply a reflection of impaired “contractility”. In fact, Ea increases in HF models of pressure overload, i.e., TAC model (52). Ventricular hypertrophy and fibrosis are hallmarks of both HFrEF and HFpEF, where the ventricular compliance is decreased as the myocardium is stiffer, which results in higher ventricular end-diastolic pressures (EDP) at any given end-diastolic volume (EDV). A ventricle with a reduced compliance would have a smaller EDV due to impaired filling for a given EDP. Nevertheless, the EDV may be very high but the EDP may not be greatly elevated when ventricular compliance increases such as in MI or other dilated cardiomyopathy with massive ventricle dilation without appreciable thickening of the wall.

## Discussion and troubleshooting

PV loop procedure can be summarized in three critical steps: (1) ensure appropriate ventilation through insertion of endotracheal tube (2) proper placement of the jugular vein catheter to infuse physiologic solution or testing drugs (3) ensure a proper placement of the PV catheter in the left ventricle.

It is important to understand differences in respiratory rate between conscious and ventilated mice in order to provide adequate ventilatory support. In fact, conscious mice have rapid shallow breaths, while ventilated mice will have much larger tidal volumes, therefore a slower respiratory rate is required to ensure appropriate alveolar ventilation. Indeed, cardiac function might be altered by respiratory acidosis (caused by too little ventilation) or respiratory alkalosis (in case of too much ventilation). This problem is empirically solved by using the lowest respiratory rate that eliminates respiratory effort from the anesthetized mouse.

Another common issue occurs following blood loss or evaporation which results in decreased blood volume with hemodynamic parameters below the normal levels. This problem is minimised by administrating fluid to the animal. Hemodynamic function can be influenced by the core body temperature which is important to maintain constant during recording through a digital feedback system. Moreover, to improve cardiac performance the anaesthetic should be adjusted during the measurement period.

Despite the complexity, of the procedure due to the small size of the mouse, PV loop analysis provides the most unique and complete information of cardiac function that cannot be obtained through any other non-invasive methodology. Therefore, LV catheterization in murine models provides an important platform for the investigation of complex pathophysiologic mechanisms that characterize cardiac disease states such as heart failure and inherited cardiomyopathies. However, microsurgical skills are necessary to successfully perform these experiments as well as for all complicated procedures that require practice.

## Concluding remarks

The greatest advantage of non-invasive methodologies for measuring cardiac function (echocardiography and MRI) is that they can be repeated in the same animal and provide direct quantification of absolute volumes and morphology. However, they are limited by their application to steady-state conditions and reliance on motion parameters that can be influenced by loading conditions and thus lack specificity to the ventricle itself. Contrarily the conductance catheter signal is proportional to volume but must be appropriately calibrated to provide accurate absolute volume measurements. Due to the high variability in the physiologic parameters reported in literature it is recommended to always use wild type control littermates to have a proper baseline as reference values. Implementation of morphological information of cardiac structure, histopathologic analysis of cardiac biopsies is always recommended to have more direct evidences for cardiac remodelling (i.e., hypertrophy, fibrosis, inflammatory cells infiltration), which represent a hallmark of heart failure.

Left ventricle catheterization requires good surgical skills of the operator, including proper endotracheal intubation for ventilator and vessels cannulation. The cost of the equipment for PV loop analysis is definitely cheaper than echocardiography machine or magnetic resonance apparatus, that still requires expert operators to run the imaging acquisition and data analysis. Moreover, PV loop analysis gives more accurate and complete information about systolic and diastolic parameters of the heart for each cardiac cycle. The major limitation of using an invasive LV catheterization procedure refers to its invasiveness, which makes this procedure not suitable for serial monitoring (contrary to echo and other non-invasive methodology). Therefore, the animal is usually sacrificed at the end of the experiment since the surgical procedure is terminal. However, PV loop analysis are suitable for evaluation of hemodynamic changes that occur in the same animal before and after drug administration.

Considering the large variability of physiological parameters observed in preclinical studies it is recommended to follow standardized guidelines regarding type of anaesthetic or detailed surgical procedure could potentially validate results across and between laboratories. This will enable the creation of a big data platform with cardiovascular physiological parameters. Clinical practice has greatly benefitted by the analysis of clinical registries (57, 58), however similar epidemiological studies in preclinical settings require more implementation and promotion. Preclinical registries for heart failure could contribute to understanding the phenotype of different models used in cardiovascular studies, or identifying new models that can be used for future cardiovascular studies. In addition, data from preclinical registry could be used to identify potential new treatments or testing the repurpose of known drugs for cardiovascular diseases. One step closer to this could be achieved by having highly specialized professional figures employed by the research institution serving as directors of preclinical cardiovascular units, responsible of coordinating collaborative studies that investigate novel mechanisms in cardiovascular pathophysiology. Another important aspect is the shortage of economic resources required to cover the cost for purchase and maintenance of equipment necessary to characterize the cardiovascular phenotype of small animals used in preclinical studies. Resources should be prioritized for groups that have established expertise in cardiac pathophysiology field, which could promote interdisciplinary collaborations with other research groups specialized in different disciplines (e.g., metabolism, diabetes, oncology, renal dysfunction, aging).
